# Compliant flooring to prevent fall-related injuries in older adults: A scoping review of biomechanical efficacy, clinical effectiveness, cost-effectiveness, and workplace safety

**DOI:** 10.1371/journal.pone.0171652

**Published:** 2017-02-06

**Authors:** Chantelle C. Lachance, Michal P. Jurkowski, Ania C. Dymarz, Stephen N. Robinovitch, Fabio Feldman, Andrew C. Laing, Dawn C. Mackey

**Affiliations:** 1 Department of Biomedical Physiology and Kinesiology, Simon Fraser University, Burnaby, British Columbia, Canada; 2 Centre for Hip Health and Mobility, University of British Columbia, Vancouver, British Columbia, Canada; 3 W.A.C. Bennett Library, Simon Fraser University, Burnaby, British Columbia, Canada; 4 Patient Safety and Injury Prevention, Fraser Health Authority, Surrey, British Columbia, Canada; 5 Department of Kinesiology, University of Waterloo, Waterloo, Ontario, Canada; San Francisco Coordinating Center, UNITED STATES

## Abstract

**Background:**

Compliant flooring, broadly defined as flooring systems or floor coverings with some level of shock absorbency, may reduce the incidence and severity of fall-related injuries in older adults; however, a lack of synthesized evidence may be limiting widespread uptake.

**Methods:**

Informed by the Arksey and O’Malley framework and guided by a Research Advisory Panel of knowledge users, we conducted a scoping review to answer: what is presented about the biomechanical efficacy, clinical effectiveness, cost-effectiveness, and workplace safety associated with compliant flooring systems that aim to prevent fall-related injuries in healthcare settings? We searched academic and grey literature databases. Any record that discussed a compliant flooring system and at least one of biomechanical efficacy, clinical effectiveness, cost-effectiveness, or workplace safety was eligible for inclusion. Two independent reviewers screened and abstracted records, charted data, and summarized results.

**Results:**

After screening 3611 titles and abstracts and 166 full-text articles, we included 84 records plus 56 companion (supplementary) reports. Biomechanical efficacy records (n = 50) demonstrate compliant flooring can reduce fall-related impact forces with minimal effects on standing and walking balance. Clinical effectiveness records (n = 20) suggest that compliant flooring may reduce injuries, but may increase risk for falls. Preliminary evidence suggests that compliant flooring may be a cost-effective strategy (n = 12), but may also result in increased physical demands for healthcare workers (n = 17).

**Conclusions:**

In summary, compliant flooring is a promising strategy for preventing fall-related injuries from a biomechanical perspective. Additional research is warranted to confirm whether compliant flooring (i) prevents fall-related injuries in real-world settings, (ii) is a cost-effective intervention strategy, and (iii) can be installed without negatively impacting workplace safety. Avenues for future research are provided, which will help to determine whether compliant flooring is recommended in healthcare environments.

## Introduction

Compliant flooring is a passive intervention approach designed to reduce the stiffness of the ground in order to attenuate the impact forces applied to the body in the event of a fall [1–5]. It has the potential to reduce the incidence and severity of all fall-related injuries caused by impact to the ground, including hip fractures and traumatic brain injuries [5]. If effective, compliant flooring should be most beneficial in high-risk environments such as long-term care (LTC), where fall and injury rates are up to three times higher than in older adults living in the community [6–10]. Yet, few LTC sites have implemented compliant flooring as a fall injury prevention strategy [5]. A barrier to uptake of compliant flooring may be the lack of synthesized evidence [11]. Indeed, a 2010 rapid report published by the Canadian Agency for Drugs and Technologies in Health [12] concluded that no relevant health technology reports, systematic reviews, meta-analyses, randomized controlled trials, non-randomized studies, or economic evaluations were found on the use of rubberized flooring in LTC to prevent or minimize fractures due to falls [12]. That report focused exclusively on the clinical effectiveness of compliant flooring in LTC. A more comprehensive review of the currently available literature on compliant flooring is required to inform fall-related injury reduction strategies in LTC and other high-risk healthcare settings. [12].

Thus, we conducted a scoping review to address the following research question: what is presented in the scientific literature about the biomechanical efficacy, clinical effectiveness, cost-effectiveness, and workplace safety associated with compliant flooring systems (as defined in Table 1) that aim to prevent fall-related injuries? These four thematic areas were deemed by the research team and the study’s Research Advisory Panel of knowledge users to be of high importance to decisions regarding uptake of compliant flooring in healthcare settings. Our specific objectives were to (i) describe the extent, range, and nature of research activity, and (ii) identify gaps in the current knowledge and directions for future research about compliant flooring. The summary of evidence in this review will be especially useful in LTC, but also applicable in acute care, assisted living, hospice, and home care environments.

**Table 1:** Key concepts and definitions pertaining to the review’s research question.

Concept	Definition
Compliant Flooring Systems	Broadly defined as flooring systems or floor coverings with some level of shock absorbency, for example, safety flooring, shock-absorbing flooring, dual stiffness flooring, rubber flooring, acoustic flooring, and carpet.
Fall-related injury	Broadly defined as fractures or soft tissue injuries (haematoma, dislocation, laceration/cut, sprain/strain, contusion/bruise, swelling, pain) as a direct result from a fall.
Biomechanical Efficacy	Evidence from experiments conducted in a controlled, laboratory environment about (i) impact force attenuation or energy absorption during real or simulated falls onto compliant flooring systems, or (ii) balance, gait and mobility performance, and/or assistive device use on compliant flooring systems.
Clinical Effectiveness	Evidence from research involving human participants and measurement of how compliant flooring systems affect fall-related injuries and falls.
Cost-effectiveness	Evidence related to the costs of compliant flooring systems relative to their effects on fall and fall-related injury healthcare costs.
Workplace Safety	Evidence about the effects of compliant flooring systems on musculoskeletal health and fatigue of healthcare workers as a direct result of differences in floor compliance.

Table reproduced from Compliant flooring to prevent fall-related injuries: a scoping review protocol, Lachance CC, Jurkowski MP, Dymarz AC, and Mackey DC, 6, e011757, 2016 with permission from BMJ Publishing Group Ltd. [11].

## Materials and methods

We developed our scoping review protocol using the 6-stage methodological framework proposed by Arksey and O’Malley (2005), and further refined by the Joanna Briggs Institute [13,14]. The study protocol has been published [11].

### Research advisory panel

We formed a Research Advisory Panel, comprised of knowledge users, to provide consultations throughout the research process. Knowledge users are individuals who are likely to use the knowledge generated by the review in order to make informed decisions about health policies, programs and/or practices [15]. The Panel included managers of fall and injury prevention for local health authorities, Directors of Care at LTC sites, a physiotherapist from LTC, representatives from LTC facilities management, and researchers with content expertise. The Panel was involved with: (i) developing the research question and key definitions integral to the review (Table 1), (ii) providing feedback on the design and implementation of the review at project meetings; (iii) helping to interpret findings and research gaps; and (iv) disseminating the review’s findings.

### Identifying relevant records

We performed academic database searches from inception to the 30^th^ of September 2015 in AgeLine, CINAHL, EBM Reviews, MEDLINE[Ovid], SportDiscus, Ergo-Abs (Ergonomic Abstracts), and Web of Science. We included records in all languages. An information scientist (ACD) oversaw development of our initial search strategy in MEDLINE[Ovid] and subsequent modifications for each database searched, according to the required syntax and search limitations of each database. We received monthly alerts from each database of all records captured in our search string that were uploaded online after our baseline search (i.e., after 30^th^ of September 2015 through to our cutoff date of 20^th^ of May 2016). Next, we searched grey literature sources to identify clinical trials, abstracts and conference proceedings, theses, dissertations and reports [11]. Additionally, we hand searched reference lists of all included articles, searched the table of contents of Age and Ageing (the journal that yielded the most included records), and consulted with content experts to identify any records not found through our other search strategies. We classified records as either academic or grey literature reports, as our academic database searches, reference list searches and consultation with content experts generated both types of literature. We imported and managed records in Refworks.

### Eligibility criteria

We included any records, published or unpublished of any study design, which described the biomechanical efficacy, clinical effectiveness, cost-effectiveness, or workplace safety of a compliant flooring system that aimed to prevent fall-related injuries (Table 1). We included a variety of rigid flooring conditions as comparators (e.g., concrete, thin vinyl, force plate). We excluded marketing materials to avoid presenting biased information and records that exclusively examined animals, children/teens (0–17 years), or athletes (all ages), since the Research Advisory Panel agreed that evidence from these populations would be unlikely to influence decisions to adopt compliant flooring in healthcare settings for older adults. Thus, we excluded records if the research was conducted within a sporting, playground, school or pediatric acute care setting. We did not limit the publication year or language of our academic database search. For feasibility reasons, we limited our grey literature search to records published in English after 1990 (i.e., when the first academic records on the biomechanical efficacy of compliant flooring were published).

### Selection of records

Two reviewers (CCL, MPJ) independently screened records from the academic database search in two levels: title and abstract screening (level 1); and full text screening of records included after level 1 screening (level 2). Pilot testing occurred before each screening stage to refine the screening spreadsheets and key definitions, which improved inter-rater agreement. The two reviewers met at regular intervals to resolve discrepancies, and involved a third reviewer (DCM) to aid with unresolved discrepancies. The third reviewer also re-screened 5% of the records excluded at level 1 to increase the rigor of screening.

For grey literature selection, we performed the screening in two stages. First, one reviewer (MPJ) searched grey literature sources for relevant records and selected all records that appeared to match the eligibility criteria [11]. Next, a second reviewer (CCL) screened the included records for any other necessary exclusion. We resolved disagreements by discussion amongst the two reviewers and involved a third reviewer (DCM) as needed.

Once all eligible records were obtained, we identified companion records by matching the authors, compliant flooring intervention (if applicable), and timeframe that the study was conducted, an approach adapted from Tricco and colleagues [16]. We designated the main record as the one which provided the most detail about the study and the companion report(s) was included for descriptive purposes only. For example, an original article would be designated as the main record while a corresponding conference abstract or review article citing the original article would be deemed a companion record.

### Data charting

Following level 2 screening, we categorized included records into the four pre-specified themes, according to their primary theme, which helped to guide the outcomes obtained during the charting process. Two reviewers (CCL and MPJ) independently charted data from all main records using a pilot-tested electronic spreadsheet. The two reviewers met after charting each theme to review entries and resolve discrepancies and involved a third reviewer (DCM) as needed. Charted information included: (1) Record summary (citation details, type of record, theme, and objectives); (2) Methods (location, setting, design, population of interest, sample, and flooring interventions and comparators); and (3) Findings (results, conclusions, limitations, and future directions).

### Collating, summarizing and reporting the results

We used numerical and narrative analyses to describe the extent, range, and nature of research activity. The numerical analysis mapped records in terms of their summary information and methods, and we reported data as number and percentage of records. The narrative analysis summarized the evidence within each of the four themes, stratified according to questions that our research team and Research Advisory Panel deemed pivotal to decision making around uptake of compliant flooring. For the narrative analysis, we coded results and conclusions of each charted record according to the question they addressed and the effect they reported (e.g., reduced impact forces, increased fall risk, etc.). In our manuscript, we use the term evidence to refer to research activity in a particular area, and more specifically, to records that described the topic of interest; we do not use evidence as a synonym for statistically significant results.

## Results

**Fig 1:**
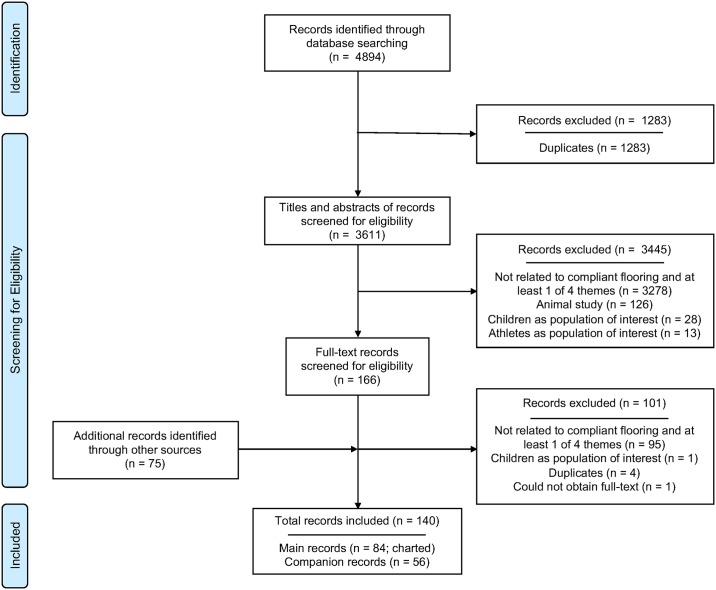
Study flow diagram. Diagram adapted from PRISMA [17]. Records identified from other sources were obtained from content experts, abstract/conference proceedings, clinical trial registries, reference lists, targeted websites, table of contents of Age Ageing, and post-baseline academic searches.

The original academic database searches conducted from inception to September 30, 2015 yielded 4894 records (Fig 1, summary of the record selection process). After removing duplicate records (n = 1283), we identified 3611 records for level 1 screening (title and abstract review). Of these, we excluded 3445 records and selected 166 for level 2 screening (full text review). After full text screening, 65 records from our academic database search reported on compliant flooring and at least one of our four themes and were thus included (101 records were excluded). Grey literature searches, hand searching techniques, consultation with content experts, and post-baseline academic searches yielded an additional 75 records. Therefore, when considering all search strategies, 140 records met our inclusion criteria. Of these, 84 main records (53 academic records, 31 grey literature records) were charted using our data charting spreadsheet, while 56 were included as companion records. We then categorized each of the 84 charted records into the four themes: biomechanical efficacy (n = 50), clinical effectiveness (n = 20), cost-effectiveness (n = 12), and workplace safety (n = 17). Fourteen records were categorized under more than one theme. Inter-rater agreement for selection of articles during level 1 and level 2 screening was good (k = 0.850, 95% CI 0.832–0.868 and k = 0.687, 95% CI 0.626–0.748, respectively). The third reviewer (DCM) who re-screened 5% of all records excluded by the two main reviewers at the level 1 screening stage, confirmed all decisions to exclude and disagreed with only one reason for exclusion (0.56% error rate). We provide the full references of all included records in S1 File.

### Numerical analysis—Description of records

#### General characteristics of included records

Table 2 summarizes the general characteristics of the 84 records included in this review, and S2 File provides our completed charting spreadsheet. The majority of records were identified through the original academic database search (n = 51, 60.7%). Most records (n = 50) were categorized under the biomechanical efficacy theme and reported on published laboratory studies. There were 20 records within the clinical effectiveness theme, 12 within cost-effectiveness, and 17 within workplace safety, with most records from these 3 themes describing research in clinical settings (e.g., acute care and LTC). Thirty-nine percent of records were retrieved from other search strategies (n = 33; 17 from consultations with content experts, 8 from conference proceedings, 5 from searching reference lists of included records, 2 from post-baseline academic searches, 1 from target websites). The majority of the included records focused primarily on compliant flooring findings (n = 67; 79.8%), which we defined as records that had at least one study objective related to compliant flooring (for primary research) or records that strictly discussed compliant flooring as a fall-related injury prevention intervention (for secondary research or non-empirical evidence).

**Table 2:** Description of included records.

Coding Category	Characteristic	Frequency	Percentage
Category (n = 84)	Academic Literature	53	63.1
	Grey Literature	31	36.9
Theme (n = 84)	Biomechanical efficacy	50	59.5
	Clinical effectiveness	20	23.8
	Workplace safety	17	20.2
	Cost-effectiveness	12	14.3
Primarily focused on compliant flooring findings (n = 84)	—	67	79.8
Source of literature (n = 84)	Record from original academic search	51	60.7
	*MEDLINE*	*21*	*41*.*2*
	*Web of Science*	*11*	*21*.*6*
	*CINAHL*	*9*	*17*.*6*
	*AgeLine*	*7*	*13*.*7*
	*Ergo-Abs*	*1*	*2*.*0*
	*Cochrane Database of Systematic Reviews*	*1*	*2*.*0*
	*SportDiscus*	*1*	*2*.*0*
	Record from experts	17	20.2
	Record from conference	8	9.5
	Record from reference list	5	6.0
	Record from academic search post-baseline	2	2.4
	*MEDLINE*	*1*	*50*.*0*
	*Web of Science*	*1*	*50*.*0*
	Record from website	1	1.2
	Record from Proquest Thesis/Dissertation search	0	0.0
	Record from clinical trial registries	0	0.0
	Record from Age Ageing table of contents	0	0.0
Journal Category (n = 53)	Engineering, other	13	24.5
	Engineering, biomedical	10	18.9
	Geriatrics and gerontology	9	17.0
	Public, environmental, and occupational health	9	17.0
	Rehabilitation	6	11.3
	Nursing	4	7.5
	Orthopaedics	2	3.8
Non-English Record (n = 84)	**—**	1	1.2
Type of Record (n = 84)	Original article	49	58.3
	Conference abstract/proceeding	13	15.5
	Non-systematic review	6	7.1
	Report	5	6.0
	Thesis/dissertation	5	6.0
	Book/book chapter	2	2.4
	Editorial/opinion piece	2	2.4
	Periodical	1	1.2
	Clinical trial registration	1	1.2
Primary Study Design (n = 86)*	Controlled experiment	51	59.3
	Simulation study	11	12.8
	Non-systematic review	7	8.1
	Randomized control trial	3	3.5
	Opinion piece	2	2.3
	Retrospective cohort study	2	2.3
	Non-randomized controlled trial	2	2.3
	Report	1	1.2
	Survey	1	1.2
	Longitudinal comparative cohort study	1	1.2
	Qualitative Methods	1	1.2
	Prospective cohort study	1	1.2
	Longitudinal observational study	1	1.2
	Not applicable	2	2.3
Country of origin (n = 85)†	United States	34	40.0
	Canada	18	21.2
	United Kingdom	14	16.5
	Sweden	6	7.1
	New Zealand	5	5.9
	Japan	3	3.5
	Australia	3	3.5
	Brazil	1	1.2
	Netherlands	1	1.2
Setting (n = 84)	Laboratory	15	17.9
	Hospital/acute care	14	16.7
	Long-term care/nursing home	10	11.9
	Community	2	2.4
	Other‡	4	4.8
	Not reported/no setting	39	46.4
Population (n = 96)	Older adults (65+ years)	34	35.4
	Adults (18–64 years)	24	25.0
	Special populations	10	10.4
	Not reported	5	5.2
	Not applicable	23	24.0
Decade of Publication (n = 84)	1980–1989	3	3.6
	1990–1999	16	19.0
	2000–2009	26	31.0
	2010–2016	39	46.4

*Notes*. Numbers may be higher than 84 records as each record can span multiple categories.

* 3 studies used both controlled experiment and modelling study designs.

^†^ 1 study took place in nursing homes across the United Kingdom and Australia.

^‡^ Other study settings include assisted living (1), hospice (1), industrial setting (1), and conference center (1).

Of the 84 included records, 53 (63.1%) were considered academic and 31 (36.9%) were grey literature. Records originated from several countries, but the majority of records were from the United States (n = 34, 40.0%), Canada (n = 18, 21.2%), and the United Kingdom (n = 14, 16.5%). Academic records came from a variety of disciplines, with most journal articles originating from engineering (43.4%), geriatrics and gerontology (17.0%), and public, environmental, and occupational health (17.0%). A majority of these academic records were original research studies (n = 49, 58.3%) that were controlled experiments. Grey literature records comprised several types of records, including conference proceedings (29.0%), theses/dissertations (16.1%), and abstracts (12.9%).

All included records were published between 1981 and May 2016, and the number of records produced per year increased steadily over time (e.g., 56.0% [47/84] of all included records were published within the last 10 years). In the first two decades of compliant flooring research (1980s and 1990s), records focused primarily on carpet and how its relative softness could prevent injury, with the exception of one record [18]. In the following decade (2000–2009), researchers investigated various combinations of carpets and underlays to create even more cushioned impact surfaces. Research on purpose-designed novel compliant flooring (NCFs; i.e., flooring systems designed specifically with the intention of preventing fall-related injuries) became more prominent in the late 2000s through present day.

#### Flooring systems examined

Across all records, we identified 183 unique flooring conditions that had been studied, which we classified into 4 categories: thick vinyl (> 5 mm; n = 2 records), carpet with no underlay (e.g., commercial grade, residential grade, carpet tile; n = 32), NCFs with no underlay or overlay (e.g., SmartCells, Sorbashock, Kradal; n = 27) and combination floorings (i.e., flooring with an overlay and an underlay which were not purchased as one single flooring type, e.g., SmartCells underlay adhered to a vinyl overlay; n = 27). Of the 32 records examining carpet conditions, 13 studied thin carpets (<0.50 cm), 8 studied thick carpets (>0.50 cm), 3 studied carpet tiles, and 11 did not report carpet thickness. Of the 27 records examining combination floorings, 15 examined vinyl with compliant flooring underlay, 3 examined vinyl with padded underlays (e.g., foam, rubber), 15 examined carpet with padded underlay, 7 examined carpet with compliant flooring underlay, and 2 examined carpet with wood underlay. Comparators or control floors studied commonly included a rigid force plate, concrete, thin vinyl, and ‘standard flooring.’

#### Consistency of terms

There were 29 unique terms used to describe NCF, which we reduced to 13 terms by allocating the stem words as minor variations (Table 3). The three most prevalent terms stemmed from derivatives of compliant flooring, safety flooring, and soft flooring.

**Table 3:** Terms used to describe compliant flooring.

Term	Number of records using term	% of records
Compliant flooring (5 minor variations*)	19	24.7
Safety flooring (3 minor variations*)	11	14.3
Soft flooring (6 minor variations*)	10	13.0
Impact absorbing flooring (2 minor variations*)	8	10.4
Energy absorbing flooring (3 minor variations*)	6	7.8
Shock-absorbing flooring	5	6.5
Low-impact flooring	5	6.5
Dual-stiffness flooring (2 minor variations*)	4	5.2
Low stiffness flooring	3	3.9
Absorptive surfaces (2 minor variations*)	3	3.9
Cushioned flooring	2	2.6
Rubberized flooring	1	1.3
New flooring system	1	1.3

*Variations of each flooring term were as follows: **Compliant flooring**—Compliant floor(ing), Compliant surface(s), more compliant floors and subfloors; Novel compliant flooring, novel compliant flooring systems; **Safety flooring**—Safety floor(ing/s), Novel safety flooring systems, Safety flooring systems; **Soft flooring**—Soft(er) floor(ing/s), Soft-surface flooring, Softer surface(s), Softer floor surfaces, Softer floor types, Softer ground; **Impact absorbing flooring**—Impact absorbent flooring, Impact-absorbing flooring; **Energy absorbing flooring**—Energy absorbing flooring, Energy-absorbing materials, Energy absorbent flooring; **Dual-stiffness flooring**–Dual-stiffness floor(ing), Dual stiffness flooring, Dually stiff floor; **Absorptive Surfaces**—Absorptive surfaces, Absorbent flooring.

### Narrative analysis—Biomechanical efficacy (n = 50)

#### What evidence exists from experiments conducted in a controlled, laboratory environment about impact force attenuation or energy absorption during real or simulated falls onto compliant flooring systems?

Twenty records provided details about the ability of compliant flooring to absorb energy and attenuate force in the event of an impact [1,3,4,18–34]. Evidence of meaningful amounts of force attenuation and energy absorption exists specifically at the hip (n = 16) [1,3,4,19–30,35], head (n = 2) [32,33], and hand [3], with most records having been conducted in a laboratory setting using artificial surrogates (e.g., headform, surrogate pelvis; n = 13, 65%), versus human subjects.

For hip impacts, carpet without an underlay did not provide enough force attenuation to suggest it would be protective against injury to the hip [3,27,28]. Carpet with the addition of an underlay (e.g., 12 mm PVC foam) provides better force attenuation than carpet alone [3,4,24,28–30], and has been shown to reduce forces below the threshold for hip fracture (2–2.5 kN) [28,30]. Compared to standard flooring, carpet with underlay has been shown to provide 7–23% force attenuation at the hip [3,21].

Overall, NCFs have been shown to attenuate hip impact forces by 16.4 to 51.2% [1,20,22] compared to a rigid surface and can reduce impact forces below the average fracture force of older cadaver femora [25]. NCFs can reduce energy absorption at the hip even more than carpet with underlay [19,23]; all NCFs tested by Glinka and colleagues (Kradal, 1.27 cm; Kradal, 2.54 cm; SmartCell, 2.54 cm with or without carpet tile overlay) absorbed 3.2 to 5.4 fold more energy than commercial carpet with foam underlay (0.96 cm) [23]. SmartCell (2.54 cm) and SofTile (10.00 cm) NCFs, were also shown to provide greater force attenuation as impact velocity increased from 2 m/s to 4 m/s (SmartCell from 17.3–33.7%; SofTile from 44.9–51.2%), suggesting these flooring systems have greater protective capacity as impact severity increases [1].

For head impacts, NCF (SmartCell, 2.54 cm with and without vinyl overlay; SofTile, 5.00 cm with and without vinyl overlay; Kradal, 1.2 cm; Kradal, 2.40 cm) provides more protection than commercial carpet [33]. Impact forces were 20–80% lower, and the authors reported that the risk of a moderate head injury (based on Head Injury Criteria) is 5–25% for a head impact on NCF versus an 80–90% risk on carpet. Additionally, Paka (2011) found that the benefits of force attenuation at the head translated into lower coup and contrecoup pressures and reduced shear stresses on the brain when falling on rubber versus wood [32].

Maki and colleagues (1990) examined hand impacts in addition to hip impacts. Contrary to other impact sites, no differences were found between carpet and standard flooring for hand impacts [3].

#### What evidence exists from experiments conducted in a controlled, laboratory environment about balance, gait and mobility performance, and/or assistive device use on compliant flooring systems?

Thirty records considered how different types of compliant flooring might affect standing or walking balance [1,18,22,23,36–61]. Human participants comprised various age groups and categories, including adults (18–64 years, n = 9), older adults (65+ years, n = 16), and special populations (n = 4), such as Parkinson’s Disease and stroke patients; 2 records did not use human participants (simulations only). Forty-eight percent tested a type of NCF (n = 13), 41% examined carpet (n = 11), and 11% looked at both carpet and NCF (n = 3). Overall, participants were able to maintain static and dynamic balance on carpet [40,41,48,49,60] and NCFs [1,22,37–39,47,50,55,57,59]. One exception was for NCFs; subjects’ anteroposterior sway range during quiet stance was higher [38] and peak center of mass displacement was larger [57] relative to rigid flooring, indicating decreased stability. Many different measures were used to test balance. For example, postural angles were measured to look at body motion [39,48], and root mean square of postural sway [1,22,37,38,50], centre of mass/pressure margin of safety [33,50,60], and mean velocity of postural sway [1,22,50,52] were measured to determine level of postural stability.

Of the 14 records that looked at gait/mobility measures, 6 did not report any significant changes when walking over carpet or NCF compared to standard flooring [18,36,43,47,51,53], 5 reported beneficial attributes of carpet or NCF on gait performance [1,42,44,54,58], and 3 reported negative attributes of carpet or NCF on gait performance [45,56,61]. Similar to the balance studies, there were a variety of measures used to measure gait and mobility performance including: gait speed [36,56,58], step length [54,58], toe clearance [44,54], Timed Up-and-Go time [51], and locomotive energy cost/loss [43,45]. A few records suggested some carpets (versus standard flooring) were favourable for walking when gait speed [58], step length [58], walking pattern [42], and obstacle avoidance [44] were considered. Willmott suggested walking on carpet was more efficient than walking on vinyl based on observed increases in gait speed [58]; however, Stephens et al. (2009) found carpet resulted in slower walking speed versus parquetry flooring [56]. Walking on rubber flooring (thickness not reported) also resulted in an increase in energy losses, but <10% in maximal angle deviations during unperturbed gait [45]. Similar to the potential benefits of carpet on gait, SmartCell NCF was suggested to be more stable for participants than vinyl; no differences were found in 6 of 8 tested gait parameters [54]. In addition, no differences were found between SmartCell (2.54 cm), SofTile (10.00 cm) and vinyl floors for Timed Up-and-Go times [1]. Only Hanger et al. (2014) suggested negative effects on gait from a low impact flooring, specifically for Parkinson’s patients [61]. Balance and gait will ultimately be impaired by large reductions in ground stiffness; however, the evidence suggests that most NCFs are stiffer than the threshold required causing significant impairment. Overall, the available evidence suggests that the compliance of these flooring systems have limited effects on gait and mobility, except in those who have neurological dysfunction.

Eight records considered assistive device use, specifically wheelchairs with carpet (n = 7) [24,31,43,62–65] and walkers with NCF (n = 2) [31,51]. The effect of compliant flooring on propelling a wheelchair largely depended on the specific floor type, but there is evidence to suggest compliant flooring may not affect the use of walkers. Van Derwoude (2003) found high-pile carpet increases rolling resistance [65] and average work per meter [62] required for individuals using wheelchairs to self-propel compared with standard flooring (e.g., tile, vinyl, and plywood). Low-pile carpet increased the energy cost and cardiopulmonary response for wheelchair locomotion when travelling from tile to carpet [43]. However, Mercer et al. (2005) did not observe any differences in manual wheelchair propulsion when comparing carpet with tile, but acknowledged that the carpet pile may have been too thin to provide enough resistance to affect propulsion [64]. In addition, Koontz et al. (2005) found that high-pile carpet, low-pile carpet, and the control surface (concrete) required less torque than interlocking pavers and a ramp condition [63]. With NCF, Hales et al. (2015) did not observe any differences in manual wheelchair propulsion when comparing proprietary compliant flooring to tile [24]. Okan et al. (2015) found that compared with standard flooring, NCF affected Timed-Up-and-Go times (a measure of mobility) in older adults who used walkers, but had no effect on self-ambulating older adults [51]. Furthermore, balance was not affected by the NCF for any participants (with or without walker use) [51]. Finally, a trial examining Powerbond compliant flooring by Yarme (2001) concluded that the flooring did not affect wheelchair and walker use for older adults residing in LTC, after 1 year of monitoring [31].

### Narrative analysis—Clinical effectiveness (n = 20)

#### What evidence exists from research involving human participants and measurement of how compliant flooring affects fall-related injuries?

Fourteen records examined whether compliant flooring would reduce the incidence of fall-related injuries. Eleven records reported some evidence of injury reduction in areas with compliant flooring versus standard flooring [4,31,34,61,66–72], 2 records reported no significant difference in the incidence of fall-related injuries [73,74], and 1 record described a protocol for an ongoing randomized controlled trial [5].

Three records provided statistical evidence that compliant flooring reduced the incidence of fall-related injuries. A 4-year retrospective cohort study in acute care found 29% fewer injuries in older patients (n = 213) on carpet (17% of falls resulted in injuries) versus vinyl (46%; unspecified type) [66]. Gustavsson and colleagues (2015) conducted a 2.5 year quasi experimental, non-randomized controlled trial to evaluate the effect of compliant flooring on fall-related injury risk for females (n = 57) in a Swedish LTC setting by comparing compliant flooring (Kradal, 1.20 cm) to standard flooring (vinyl, linoleum, ceramic tile, thickness not specified) [67]. The injury/fall rate was 30.3% on standard flooring and 16.9% on compliant flooring [67]. After adjusting for body-mass index, compliant flooring significantly reduced the relative risk of injury in the event of a fall by 59% (RR 0.41; 95% Cl 0.20–0.80) compared to standard flooring [67]. A 2-year prospective cohort study in LTC aimed to evaluate whether floor properties had a significant effect on the risk of a fracture occurring in a fall (sample size not reported). By examining different flooring types (uncarpeted concrete, carpeted concrete, uncarpeted wood, carpeted wood—thickness not specified for any floors), the authors estimated that the risk of breaking a hip in a fall would be reduced by 80% if carpet was laid on uncarpeted wooden floors (OR 1.78; 95% CI 1.33–2.35) [4]. Carpet with a concrete underlay was not associated with a significantly lower risk of hip fracture following a fall. In addition, 7 grey literature records and 1 non-systematic review provided general statements that compliant flooring could reduce the incidence of injuries (sample sizes not reported), but did not provide sufficient details to conclude whether this evidence was from measured, experimental observation (i.e., no quantitative results were presented nor was any statistical significance testing performed) [30,33,60,75–79].

Among the 2 records that reported no significant difference in the incidence of fall-related injuries, Warren and Hanger (2012) conducted a non-randomized, longitudinal observational study (n = 4641) in acute care to examine fracture rates on carpet (loop-pile carpet tile, 0.50 cm) versus thick vinyl (unspecified type, 0.50 cm) [74]. 15 fractures occurred on carpet (0.75 fractures per 100 falls) compared to a non-statistically different 11 that occurred on vinyl (1.33 per 100 falls) [74]. In an unblinded, pilot cluster randomized controlled trial conducted by Drahota and colleagues, geriatric wards at eight hospitals were allocated to compliant (Tarkett Omnisports EXCEL, 0.83 cm) or standard (control, unspecified type) flooring (Helping Injury Prevention in Hospitalised Older People (HIP-HOP) Flooring Study; n = 442) [73]. During one year of outcome monitoring, 23% of falls (8 of 35) were injurious in intervention wards compared to 42% of falls (14 of 33) in control wards. Though this difference was non-significant due to an insufficient sample size (IRR = 0.58, 95% CI: 81% reduction– 91% increase), the authors concluded that shock-absorbing flooring could potentially reduce injury rates by 42%. No moderate or major severity injuries occurred in intervention wards, while six occurred in control wards [73].

The protocol record describes the Flooring for Injury Prevention (FLIP) Study, which is a 4-year, parallel group, randomized controlled superiority trial [5]. Outcome ascertainment began September 2013. One-hundred and fifty resident rooms at one LTC site were randomized to receive either NCF (SmartCells, 2.54 cm; 74 rooms) or standard flooring (plywood, 2.54 cm; 76 rooms), each with identical vinyl (0.20 cm) floor covering. The primary outcome is to determine whether NCF reduces serious fall-related injuries relative to control flooring, defined as any impact-related injury due to a fall in a study (resident) room that results in Emergency Department visit or hospital admission. The trial is also monitoring the incidence of minor fall-related injuries, fractures and falls, as well as the number of fallers, and healthcare utilization and costs for serious fall-related injuries [5].

In addition, two records discussed severity of fall-related injuries, though neither record performed statistical testing on the reported frequency counts [67,80]. A 2.5-year retrospective study at a LTC site found there was a non-significant trend for fewer bruises (66%) and abrasions (56%) from falls on compliant flooring (SmartCells, 2.54 cm with vinyl overlay) than falls on standard flooring; however, there was a higher prevalence of redness (100%) and cuts (43%) on compliant flooring [80]. While two falls on standard flooring resulted in fracture, no falls on compliant flooring resulted in fracture [80]. Though Gustavsson et al. (2015)’s study reported a significant 59% reduction in risk for fall-related injury for falls that occurred on compliant flooring (Kradal, 1.25 cm), they also acknowledged that 80% of fall-related injuries were of minor severity (e.g., distinct pain, bruising, swelling) [67]. While there was a significant reduction in minor and moderate injuries, there was insufficient statistical power to test the effect of compliant flooring on less common, more serious fall-related injuries [67].

#### What evidence exists from research involving human participants and measurement of how compliant flooring affects falls?

Three records performed statistical testing to examine whether compliant flooring increases the risk of falling in older adults [4,73,74]. Two records, which described studies in acute care, did not find evidence that compliant flooring alters rates of falling [73,74]. Firstly, Warren et al. (2012) did not find a significant difference in fall rates following a change from carpet tile (0.50 cm) to vinyl (0.50 cm) [74]. There were 854 falls on carpet in the 12 months prior to the flooring change and 878 falls on vinyl the 12 months after (19.5 and 19.6 falls/1000 bed days, respectively) [74]. Further, in the Drahota et al. study, the incident rate for falls was only slightly higher in the intervention group (n = 35 falls; IR = 7.81 falls per 1,000 OBD) compared with control (n = 33 falls; IR = 7.17 falls per 1,000 OBD), since there were more recurrent fallers in the control group [73]. The (uncertain) estimated effect of the intervention flooring on falls was an increase of ~7% relative to control (adjusted IRR = 1.07, 95% CI = 0.64–1.81, k = 0.226), which they found to be larger when examining hazard ratios (adjusted HR = 1.13, 95% CI = 0.83–1.55) [73]. Finally, the Simpson et al. study (the same prospective cohort study that found an 80% hip fracture risk reduction if carpet was laid on uncarpeted wooden flooring) found an increase in the rate of falling on carpet (RR 2.74–4.30) [4], though the authors mentioned that these results were likely confounded by differences in exposure time.

### Narrative analysis—Cost effectiveness (n = 12)

#### What evidence exists related to the costs of compliant flooring systems relative to their effects on fall and fall-related injury healthcare costs?

Six records discussed the direct cost [1,75–77,81] or incremental cost of purchasing and installing NCFs relative to standard flooring [1,76–78,81] (Table 4). Costs have been reported for the following brands: SmartCell, SoftTile, Tarkett Omnisport EXCEL, Kradal, and Penn State Flooring. Acknowledging that the pricing of compliant flooring was reported using different currencies and over multiple years, we converted all costs to reflect 2015 US dollars [79]. The average absolute direct cost of purchasing and installing a NCF system was $236.61 US dollars per square meter (range: $100.54 –$538.20 per square meter) [1,75–77,81]. In addition, the average incremental cost of purchasing and installing a NCF system was $196.30 US dollars per square meter (range: $50.27 –$511.29). Two records described but did not quantify that carpet was more expensive to install, maintain and replace compared to vinyl [24,34].

**Table 4:** Costs of novel compliant flooring systems

Citation	Verbatim cost of compliant flooring	Converted cost (2015 USD/m^2^)	Brand of Compliant Flooring	Verbatim incremental cost†	Converted incremental cost (2015 USD/m^2^)
Laing (2009)	161 CAD/m^2^	$121.14	SmartCell or SofTile	134 CAD	$100.83
Lange (2012)	1600 SEK/m^2^	$182.34	Not reported	1200 SEK	$136.75
Latimer (2013)	164 GBP/m^2^	$240.82	Tarkett Omnisport EXCEL	Not reported	N/A
Njogu (2008)	150 NZD/m^2^	$100.54	Not reported	75 NZD	$50.27
Ryen (2015)	Not reported	N/A	Kradal	1600 SEK	$182.34
Zacker (1998)	50 USD/ft^2^	$538.20	Penn State Flooring with vinyl overlay	47.50 USD	$511.29

*Notes*. Acknowledging that the pricing of flooring came from many years, if this was based from 2015 prices, this table would reflect the 2015 values in US Dollars. Source. https://www.irs.gov/individuals/international-taxpayers/yearly-average-currency-exchange-rates; Incremental cost = cost of compliant flooring—cost of traditional flooring; /m2 = per square meter; /ft2 = per square foot; CAD = Canadian Dollar; SEK = Swedish Krona; GBP = British Pound; NZD = New Zealand Dollar; USD = US Dollar

Six records provided cost-effectiveness estimates for compliant flooring [1,24,75–78,81]. The 2 most extensive studies of cost-effectiveness, which were published in peer-reviewed journals, examined Tarkett Omnisport EXCEL (0.83 cm) [75] and Penn State Flooring (2.50 cm + vinyl overlay) [79]. As part of the HIP-HOP Flooring Study [73], Latimer and colleagues reported model estimate costs and quality adjusted life-years (QALYs) of $57 415.57 USD and 0.425 per patient in the control group and $56 177.68 USD and 0.419 in the intervention group. They concluded that Tarkett Omnisport EXCEL flooring is associated with a cost reduction of $1 237.89 USD per patient, a QALY loss of 0.006 and an incremental cost-effectiveness ratio (ICER) of $198 095.45 USD [75]. The authors suggested compliant flooring is cost-effective as the cost savings per QALY lost are >$29 368.58 USD while acknowledging their primary analysis results were extremely sensitive to the risk of falling. Thus Latimer and colleagues performed a secondary analysis that assumed an equal risk of falls, but lower proportion of severe falls in the intervention group (as suggested by the associated clinical trial [73]), to find that the intervention floor was expected to provide cost savings and QALY gains, making it a dominant strategy. Another cost-effectiveness analysis by Zacker and Shea (1998), estimated a payback period of 10.5 years if only direct costs avoided were evaluated (cost-benefit ratio (CBR) = 0.61) and approximately 11 months when direct and indirect costs were included (CBR = 0.06) [77]. They derived these numbers using the following assumptions: five falls per year for eight patients, the direct cost of a hip fracture being $18 000 (USD) and the expected costs of falls with conventional flooring as $14 400 per year versus $7 200 with safety flooring [77]. For the best case, the CBR was very favourable and the cost-effectiveness ratio (CER) was less than $0 per life-year saved. This result was robust in the worst-case scenario when considering indirect costs. However, when only direct costs were considered in this case, the CBR was >1 and no longer faourable. Zacker and Shea (1998) concluded that the flooring system could be cost saving given the expected reduction in hip fractures.

### Narrative analysis—Workplace safety (n = 17)

#### What evidence exists from research about the effects of compliant flooring systems on musculoskeletal health and fatigue of healthcare workers as a direct result of differences in flooring compliance?

Of the 17 articles that examined workplace safety of compliant flooring, five original research studies [34,61,82–84] found benefits of compliant flooring for healthcare workers. Survey results from 2 records suggested that compliant flooring (unspecified type) provides more comfort for acute care staff [51,61] (sample sizes: not reported; n = 9) than standard (unspecified type) flooring. In addition, a 42-week longitudinal comparative cohort study found that carpet (tufted-level loop carpet tile, thickness not reported) and compliant flooring (resilient rubber, 0.30 cm) reduced perceptions of fatigue due to underfoot impact in acute care staff compared to control (Terrazzo) flooring [83] (n = 102). A non-randomized controlled trial of 153 LTC staff observed the effects of installing a thick vinyl (0.40 cm) compared to a thin vinyl (0.20 cm) [82]. After a 2-year follow-up, LTC staff reported decreased pain intensity score in their feet (mean difference -1.67, 95% CI– 2.70 to– 0.65) [82]. Finally, one record found that carpet increased the forces required for nurses and nursing students to push a medicine cart (*see risks section)*, but reduced the amount of force required to stop the cart compared to tile flooring [84].

Most (n = 16, 88.9%) of the workplace safety records reported some negative effects of compliant flooring on healthcare workers, including 14 original research studies [5,51,61,65,73,83–91] and two opinion pieces [92,93]. Focus group findings (sample size not reported) revealed that compliant flooring (Kradal, 1.20 cm) increased subjective ratings of leg fatigue for LTC nurses and difficulty when maneuvering equipment when compared to standard flooring (vinyl, linoleum, tile) [89]. In addition, six other records noted increased subjective ratings of perceived fatigue when maneuvering equipment, including beds [51,61,87,89,92,93], wheelchairs [51,89,93], stretchers [92,93], and floor-based lifts [51,61,87,89,94] over both carpet (n = 3 records) and NCF (n = 4 records) in acute and LTC settings.

Eight records revealed that carpet and NCFs increase the forces required to maneuver (push, pull, and/or turn) carts [84–86], beds/patient trolleys [90], wheelchairs [28,65], and floor-based lifts [90,91,94]. When considering recommended limits to safely maneuver equipment, 3 (of 6) records that examined carpet [86,91,94] and 2 (of 3) records that examined NCF [90,94] recorded values that were over the recommended limits, suggesting an increased risk for injury. Keeping the forces required to move equipment within recommended limits is essential to prevent injury, as indicated by one record which documented 5 adverse events from staff working on a NCF in a 16 month period, including 1 lower back muscle strain while moving a patient on a trolley (Tarkett Omnisport Excel, 0.83 cm) [73].

## Discussion

This study is the first scoping review to synthesize the available evidence about the biomechanical efficacy, clinical effectiveness, cost-effectiveness, and workplace safety associated with compliant flooring systems that aim to prevent fall-related injuries. We followed the original Arksey and O’Malley framework (2005) and published updates to it, and we searched both academic and grey literature. In addition, our Research Advisory Panel of knowledge users provided guidance on development of the research question, key definitions, interpretation of findings and gaps in the research, and dissemination of findings. This scoping review builds considerably on a 2010 report on compliant flooring by the Canadian Agency for Drugs and Technologies in Health [12].

Eighty-four records plus 56 companion records satisfied our inclusion criteria. Our included records comprised 183 unique flooring conditions, which we categorized into thick vinyl (> 5 mm), carpet, NCFs, and combination floorings, and 29 unique terms to describe NCFs. The majority of records were identified through our original academic database search (60.7%); however, 39.3% came from other approaches, emphasizing the importance of additional search strategies.

The primary finding of this review is that compliant flooring is a promising strategy for fall injury prevention from a biomechanical perspective, but more research is needed in clinical environments to determine if the established biomechanical efficacy translates into an injury prevention strategy that is clinically and cost-effective and that does not negatively influence safety in the workplace. Though preliminary evidence exists, there is a paucity of literature on these 3 themes (clinical effectiveness n = 20, cost-effectiveness n = 12, workplace safety n = 17) compared to the biomechanical efficacy theme (n = 50).

The biomechanical efficacy theme contained the largest number of records and examined the greatest number of compliant flooring systems. Laboratory evidence demonstrates compliant flooring reduces impact forces during simulated falls and has minimal effects on standing or walking balance for self-ambulating individuals. However, preliminary evidence suggests that balance and mobility performance may be impaired for users of assistive devices (e.g., wheelchairs). Despite the substantive biomechanical evidence, sample sizes of individual studies were generally small and testing protocols used surrogate hips, heads, hands or young adults as participants. Thus, results may or may not be transferrable to target users, including older adults at risk of falling. Overall, results from biomechanical testing of compliant flooring provided strong enough evidence that 8 records suggested a clinical trial is warranted to test clinical effectiveness in a real-world setting [1,3,19,22,24,28,33,59].

A small number of clinical studies have examined the ability of compliant flooring to prevent fall-related injuries in acute and LTC settings. Research performed in clinical settings provided some preliminary, but not conclusive, evidence of compliant flooring reducing the incidence and severity of fall-related injuries in such settings, but also indicated that compliant flooring may increase the risk of falling. The number of fall-related injuries observed within individual studies have been small, precluding definitive conclusions. In particular, no randomized controlled trial has been sufficiently powered to test the effectiveness of compliant flooring for reducing fall-related injuries. However, the FLIP Study is an ongoing randomized controlled trial in LTC, and it has been powered for the primary outcome of serious fall-related injury [5].

Overall, compliant flooring systems cost more than standard flooring. The average reported 2015 cost of NCF was $236.61 US dollars per square meter, and the average 2015 incremental cost relative to standard flooring was $196.30 US dollars per square meter. The records examining cost-effectiveness provide preliminary indications that compliant flooring may be a cost-effective strategy for healthcare systems with older adults at risk for falling. Most cost-effectiveness analyses were based on the potential cost savings from hip fracture prevention only.

From the current body of evidence, compliant flooring may pose health and safety risks for healthcare workers. Although some evidence found compliant flooring increased comfort and reduced the perception of fatigue due to underfoot impact among healthcare workers, more evidence suggested that compliant flooring makes it more difficult for workers to perform standard tasks, including maneuvering equipment (e.g., floor-based lifts, wheelchairs, beds/patient trolleys), compared to standard flooring.

### Gaps in the literature and recommendations for future research

In conducting this review, we identified a number of gaps in the available evidence that suggest important avenues for future research. First, the highest priority is for additional studies to investigate the clinical effectiveness studies of compliant flooring. Such studies require rigorous methodologies, such as blinded, randomized controlled trials with sufficient sample sizes and follow-up periods to provide adequately powered and conclusive results. To help facilitate future cost-effectiveness studies, future clinical trials should consider including economic evaluations. Additionally, since so many falls are unwitnessed, researchers should consider video surveillance of study areas to increase accuracy in reporting falls and fall-related injuries [95], though privacy issues will need to be addressed in bedroom and bathroom areas. When study sites have multiple types of flooring, it would be beneficial to track participant exposure time to compliant flooring to yield more accurate estimates of the effects of compliant flooring. Future studies should also consider evaluating whether compliant flooring results in an increased risk for falling and determine if specific brands of compliant flooring have more or less of an effect on falling than others. No clinical studies in this review discussed where to prioritize the installation of compliant flooring within a LTC site (i.e., coverage), yet this is a practical issue that knowledge users may encounter, especially when under financial constraints.

Second, most cost-effectiveness studies have only included hip fracture costs; thus, there is value in expanding to include other injuries (e.g., all fractures, head injuries, soft-tissue injuries) in future research. Since there were relatively few records examining cost-effectiveness, future research should examine different types of compliant flooring to determine if certain brands of compliant flooring are more cost-effective than others. In addition, although some records mentioned the cost differences between standard and compliant flooring systems, there was no mention of how costs would vary if compliant flooring was installed during a building retrofit installation versus a new build. Thus, researchers should consider costing out the differences between these two scenarios to better inform knowledge users.

Third, the number of records representing the workplace theme is relatively small and, in general, conclusions were derived from small sample sizes. Despite several records acknowledging that compliant flooring increases the effort or forces required for participants to use medical equipment, only a few records tested a proposed solution (i.e., modifications to wheel characteristics, using motorized floor-based and ceiling-mounted patient lift systems instead of traditional floor-based lifts [85,89,91,94]). Future workplace safety studies should work to identify and examine additional potential solutions to the previously identified concerns of maneuvering equipment over compliant flooring. Future study protocols should also reflect tasks and situations that are common in the workplace, such as measuring the force requirements to move equipment in confined spaces [91,94], testing a variety of healthcare worker populations, including novice and aging populations [84,87,89,92], randomizing the conditions being compared, and blinding the flooring systems that are being evaluated (when possible) [51].

Finally, despite the promising existing biomechanical evidence, future biomechanical studies should consider examining the effects of compliant flooring on dynamic balance and more complex mobility tasks (e.g., gait performance while conducting activities of daily living). It is especially important to test these outcomes, and other biomechanical measures, using the population of interest (i.e., frail older adults), as there is a dearth of biomechanical studies with older adults who are at high-risk of falling or having a history of falling. Most biomechanical records with human subjects involved relatively young adults (18–64 years) or healthy, community-dwelling older adults (65+ years) to draw conclusions about how compliant flooring may affect older adults in general. For example, only 19.6% (n = 10) of the records in this theme involved high-risk older adults who were not living independently [31,47,50,61,96–101], and only 13.7% (n = 7) examined special populations [64,96,102–104] that are common in high-risk environments (e.g., LTC), such as individuals with Parkinson’s disease or those whose mobility is dependent on an assistive device. Understanding the effects of compliant flooring on various populations will help to assess feasibility of installing compliant flooring in high-risk environments, including acute care and LTC.

### Limitations

Though our scoping review followed a standardized approach and used a Research Advisory Panel as consultants throughout the research process, our review has certain limitations. The review provided breadth but not depth about the topic, consistent with scoping review methodology [13]. Additionally, we did not assess the risk of bias nor use a rating of quality of evidence, and therefore, we cannot grade recommendations for practice [14]. This is also consistent with scoping review methodology [14]. An additional limitation is that the grey literature search was limited to records published in the English language from 1990 or later, which is when the first academic records on the biomechanical efficacy of compliant flooring were published. Finally, the results reported in the records we examined were based on the specific flooring types used in each study, and therefore, may not be generalizable to other floors.

## Conclusions

In conclusion, compliant flooring is a promising strategy for fall injury prevention based on existing literature that has examined biomechanical efficacy, clinical and cost-effectiveness and workplace safety. This scoping review responds to the information needs of healthcare decision makers tasked with preventing fall-related injuries by synthesizing the available evidence about compliant flooring as a potential intervention for preventing fall-related injuries in older adults. We have identified gaps in the available evidence and have suggested avenues for future research.

## Supporting information

S1 File S1 list of included records. (DOCX)

S2 File S2 charting spreadsheet. (XLSX)
