# Supplementary material for: Compliant flooring to prevent fall-related injuries in older adults: A scoping review of biomechanical efficacy, clinical effectiveness, cost-effectiveness, and workplace safety
Source: PLoS One. 2017 Feb 6;12(2):e0171652. doi: 10.1371/journal.pone.0171652 (PMC5293217; doi:10.1371/journal.pone.0171652)
Supplement: S1 File — (DOCX) [file pone.0171652.s001.docx]

**Supplementary File 1**

**List of References of Included Records**

1. Adrian M, Deustch H, Riccio GE. Walking patterns and falling in the elderly. 1st ed. Department of Kinesiology, University of Illinois at Urbana-Champaign; 1990.
2. Al-Eisawi KW, Kerk CJ, Congleton JJ, Amendola AA, Jenkins OC, Gaines W. Factors affecting minimum push and pull forces of manual carts. Appl Ergon. 1999;30(3):235–45.
3. Australian New Zealand Clinical Trials Registry: Sydney (NSW): NHMRC Clinical Trials Centre, University of Sydney (Australia); 2005 - Identifier ACTRN12614000757617 [Internet]. Exploratory study of ease of walking for Parkinson’s and stroke patients (compared to control patients) on low impact flooring; 2014 Jul 17 [cited 2016 Feb 22]; [5 pages]. Available from: https://www.anzctr.org.au/Trial/Registration/TrialReview.aspx?id=366684.
4. Beach R. Effect of compliant flooring on postural stability in an older adult population and in individuals with Parkinson’s disease [Internet]. Doctoral dissertation, University of Datyon; 2013. Available from: https://etd.ohiolink.edu/!etd.send_file?accession=dayton1385131135&disposition=inline.
5. Beach R, Jackson K, Bigelow KE. Effect of compliant flooring on postural stability in an older adult population. In: Proceedings of the 37th Annual Conference of the American Society of Biomechanics; 2013 Sep 4-7 [Internet]. Omaha, Nebraska, USA; Available from: http://www.asbweb.org/conferences/2013/abstracts/342.pdf.
6. Bhan S, Levine IC, Laing AC. Energy absorption during impact on the proximal femur is affected by body mass index and flooring surface. J Biomech. 2014;47(10):2391–7.
7. Bhan S, Levine I, Laing AC. The influence of body mass index and gender on the impact attenuation properties of flooring systems. J Appl Biomech. 2013;29(6):731–9.
8. Biman D, Wimpee J, Das B. Ergonomics evaluation and redesign of a hospital meal cart. Appl Ergon. 2002;33(4):309–18.
9. Birge SJ. Osteoporosis and hip fracture. Clin Geriatr Med. 1993;9(1):69–86.
10. Boyer J, Lin JH, Chang CC. Description and analysis of hand forces in medicine cart pushing tasks. Appl Ergon. 2013;44(1):48–57.
11. Brawley EC. Environment- A Silent Partner in Caregiving. In: Kaplan M, Hoffman SB, editors. Behaviors in dementia: Best practices for successful management. Health Professions Press; 1998. p. 107–24.
12. Buschman J, Leitkam S, Bush TR. Compliant flooring and its effects on posture: Differences between young and older populations. In: Proceedings of the 40th Annual Conference of the American Society of Biomechanics ; 1998 Aug 5-8 [Internet]. Columbus, Ohio, USA. p. 943-4; Available from: http://www.asbweb.org/2015-annual-conference-columbus-oh.
13. Casalena, J. A., Ovaert, T. C., Cavanagh, P. R., & Streit, D. A. (1998). The Penn State Safety Floor: Part I - Design parameters associated with walking deflections. Journal of Biomechanical Engineering, 120(4), 518–26. Retrieved from http://www.ncbi.nlm.nih.gov/pubmed/10412423.
14. Chesney DA, Axelson PW. Preliminary test method for the determination of surface firmness [wheelchair propulsion]. IEEE Trans Rehabil Eng [Internet]. 1996;4(3):182–7. Available from: http://ieeexplore.ieee.org/lpdocs/epic03/wrapper.htm?arnumber=536773.
15. Cogswell L. A question for readers: Carpets in hospitals. Am J Nurs. 2005;105(7):16.
16. Cummings SR, Melton LJ. Osteoporosis I: Epidemiology and outcomes of osteoporotic fractures. Vol. 359, Lancet. 2002. p. 1761–7.
17. Dickinson JI, Shroyer JL, Elias JW, Hutton JT, Gentry GM. The effect of selected residential carpet and pad on the balance of healthy older adults. Environ Behav [Internet]. 2001;33(2):279–95. Available from: http://eab.sagepub.com/cgi/doi/10.1177/00139160121972990.
18. Dickinson JI, Shroyer JL, Elias JW. The influence of commercial-grade carpet on postural sway and balance strategy among older adults. Gerontologist [Internet]. 2002;42(4):552–9. Available from: http://www.ncbi.nlm.nih.gov/pubmed/12145383.
19. Donald IP, Pitt K, Armstrong E, Shuttleworth H. Preventing falls on an elderly care rehabilitation ward. Clin Rehabil. 2000;14(2):178–85.
20. Drahota AK, Kward D, Udell JE, Soilemezi D, Ogollah R, Higgins B, et al. Pilot cluster randomised controlled trial of flooring to reduce injuries from falls in wards for older people. Age Ageing. 2013;42(5):633–40.
21. Finlay O, Beringer T. Effects of floor coverings on measurement of gait and plantar pressure. Physiotherapy. 2007;93(2):144–50.
22. Gardner TN, Simpson AHRW, Booth C, Sprukkelhorst P, Evans M, Kenwright J, et al. Measurement of impact force, simulation of fall and hip fracture. Med Eng Phys. 1998;20(1):57–65.
23. Glaser RM, Sawka MN, Wilde SW, Woodrow BK, Suryaprasad AG. Energy cost and cardiopulmonary responses for wheelchair locomotion and walking on tile and on carpet. Paraplegia [Internet]. 1981;19(4):220–6. Available from: http://www.ncbi.nlm.nih.gov/pubmed/7290731.
24. Glinka MN, Cheema KP, Robinovitch SN, Laing AC. Quantification of the trade-off between force attenuation and balance impairment in the design of compliant safety floors. J Appl Biomech. 2013;29(5):563–72.
25. Glinka MN, Karakolis T, Callaghan JP, Laing AC. Characterization of the protective capacity of flooring systems using force-deflection profiling. Med Eng Phys. 2013;35(1):108–15.
26. Gustavsson J, Bonander C, Andersson R, Nilson F. Investigating the fall-injury reducing effect of impact absorbing flooring among female nursing home residents: Initial results. Inj Prev [Internet]. 2015;21:320–4. Available from: http://injuryprevention.bmj.com/content/early/2015/04/01/injuryprev-2014-041468.short?g=w_injuryprevention_ahead_tab.
27. Gustavsson J. Working in a nursing home with Impact Absorbing Flooring: A qualitative study on the experiences of licensed practical nurses [Internet]. Doctoral Dissertation, Karlstad University; 2015. Available from: http://www.diva-portal.org/smash/record.jsf?pid=diva2:865326&dswid=-440.
28. Hales M, Johnson JD, Asbury G, Evans N. Influence of floor covering composition on force attenuation during falls, wheelchair mobility, and slip resistance. AATCC Rev [Internet]. 2015;15(6):44–53. Available from: http://openurl.ingenta.com/content/xref?genre=article&issn=1532-8813&volume=15&issue=6&spage=44.
29. Hanger HC, Hurley K, Hurring S, White A. Low Impact flooring - is it practical in a hospital? In: Proceedings of the 6th Biennial Australia and New Zealand Falls Prevention Conference. Sydney, Australia; 2014. p. 66.
30. Harris DD. The influence of flooring on environmental stressors: A study of three flooring materials in a hospital. Heal Environ Res Des J. 2015;8(3):9–29.
31. Healey F. Does flooring type affect risk of injury in older in-patients? Nurs Times [Internet]. 1994;90(27):40–1. Available from: http://www.scopus.com/inward/record.url?eid=2-s2.0-0028766773&partnerID=tZOtx3y1.
32. Heijnen M, Rietdyk S. Failure to clear stationary, visible obstacles is affected by surface characteristics. In: Proceedings of the 22nd International Society of Posture and Gait Research World Congress. Vancouver, British Columbia, Canada; 2014.
33. Hernandez ME, Ashton-Miller J. Effect of surface compliance on stepping responses to trunk perturbations. In: Proceedings of the 31st Annual Conference of the American Society of Biomechanics; 2007 Aug 22-25 [Internet]. Palo Alto, California, USA; Available from: http://www.asbweb.org/blog/2013/09/12/2007-annual-meeting.
34. Hester AL. Preventing injuries from patient falls. Am Nurse Today. 2015;10(7):9–12.
35. Kleiner AFR, do Carmo AA, Sueyoshi P, de Barros RML. Ground reaction forces and friction during stroke gait: The flooring surface effect. In: Proceedings of the 24th Biennial Congress of the International Society of Biomechanics; 2013 Aug 4-9 [Internet]. Natal, Brazil; Available from: https://isbweb.org/images/conferences/isb-congresses/2013/oral/cb-cp-stroke-spasticity.06.pdf.
36. Knoefel FD, Mousseau M, Berry M. Pilot study to assess mobility safety on a dual-stiffness floor. Can J Geriatr. 2008;11(2):110–2.
37. Knoefel F, Patrick L, Taylor J, Goubran R. Dual-stiffness flooring: Can it reduce fracture rates associated with falls? J Am Med Dir Assoc. 2013;14(4):303–5.
38. Koontz AM, Cooper RA, Boninger ML, Yang Y, Impink BG, van der Woude LH. A kinetic analysis of manual wheelchair propulsion during start-up on select indoor and outdoor surfaces. J Rehabil Res Dev. 2005;42(4):447–58.
39. Kuwae Y, Yuji T, Higashi Y, Fujimoto T, Sekine M, Tamura T. The influence of floor material on standing and walking by hemiplegic patients. In: Proceedings of the 26th Annual Conference of the IEEE Engineering in Medicine and Biology; 2004 Sep 1-5 [Internet]. San Francisco, California, USA; p. 4770–2. Available from: http://ieeexplore.ieee.org/stamp/stamp.jsp?tp=&arnumber=1404320.
40. Lachance CC, Feldman F, Laing AC, Leung PM, Robinovitch SN, Mackey DC. Study protocol for the flooring for injury prevention (FLIP) study: A randomised controlled trial in long-term care. Inj Prev [Internet]. 2016;1–8. Available from: http://www.ncbi.nlm.nih.gov/pubmed/27044272.
41. Lachance CC, Korall AMB, Russell CM, Feldman F, Robinovitch SN, Mackey DC. External hand forces exerted by long-term care staff to push floor-based lifts: Effects of flooring system and resident weight. Hum Factors [Internet]. 2016; Available from: http://hfs.sagepub.com/content/early/2016/04/19/0018720816644083.long.
42. Lackey SP, Wilik E, Psencik D, Sauer S, Becker A. A question of carpeting. Am J Nurs. 2005;105(10):16.
43. Laing AC, Robinovitch SN. Low stiffness floors can attenuate fall-related femoral impact forces by up to 50% without substantially impairing balance in older women. Accid Anal Prev. 2009;41(3):642–50.
44. Laing AC, Tootoonchi I, Robinovitch SN. Design of compliant floors to reduce impact forces during falls on the hip. In: Journal of Bone and Mineral Research. 2004. p. S315.
45. Lange B. The impact of absorbent floor in reducing hip fractures: A cost-utility analysis among institutionalized elderly. Master’s Thesis, Karlstad University; 2012.
46. Latimer N, Dixon S, Drahota AK, Severs M. Cost-utility analysis of a shock-absorbing floor intervention to prevent injuries from falls in hospital wards for older people. Age Ageing. 2013;42(5):641–5.
47. Li N, Tsushima E, Tsushima H. Comparison of impact force attenuation by various combinations of hip protector and flooring material using a simplified fall-impact simulation device. J Biomech. 2013;46(6):1140–6.
48. Lord SR, Clark RD, Webster IW. Visual acuity and contrast sensitivity in relation to falls in an elderly population. Age Ageing [Internet]. 1991;20(3):175–81. Available from: http://ageing.oxfordjournals.org/cgi/doi/10.1093/ageing/20.3.175.
49. Ma C. The effects of safety flooring on sit-to-stand and quiet stance balance reactions in retirement home-dwellers. Master’s Thesis, University of Waterloo; 2012.
50. Maki BE, Fernie GR. Impact attenuation of floor coverings in simulated falling accidents. Appl Ergon. 1990;21(2):107–14.
51. Marras WS, Knapik GG, Ferguson S. Lumbar spine forces during manoeuvring of ceiling-based and floor-based patient transfer devices. Ergonomics. 2009;52(3):384–97.
52. McGill SM, Callaghan JP. Impact forces following the unexpected removal of a chair while sitting. Accid Anal Prev. 1998;31(1-2):85–9.
53. Mercer J, Boninger M, Koontz AM, Pearlman J, Cooper R. Kinetic analysis of manual wheelchair propulsions over three surfaces. In: Proceedings of the 29th Annual Meeting American Society of Biomechanics; 2005 Jul 31-Aug 5. Cleveland, Ohio, USA; p. 169.
54. Minns J, Nabhani F, Bamford JS. Can flooring and underlay materials reduce hip fractures in older people? Nurs Older People. 2004;16(5):16–20.
55. Minns J, Tracey S. Wheelchair pushing forces over a vinyl and a new shock-absorbing flooring. Br J Occup Ther. 2011;74(1):41–3.
56. Nabhani F, Bamford J. Mechanical testing of hip protectors. J Mater Process Technol. 2002;124(3):311–8.
57. Nabhani F, Bamford JS. Impact properties of floor coverings and their role during simulated hip fractures. J Mater Process Technol. 2004;153-154(1-3):139–44.
58. Njogu F, Brown P. Cost effectiveness of impact absorbent flooring in reducing fractures among institutionalized elderly. Center for Health Services Research Policy. Auckland, New Zealand; 2008.
59. Okan J. Development of a fall-injury reducing flooring system in geriatric care with focus on improving the models used in the biomechanical simulations and evaluating the first test area [Internet]. Master’s Thesis, KTH Royal Institute of Technology; 2015. Available from: http://www.diva-portal.org/smash/record.jsf?dswid=1236&pid=diva2:872966&c=1&searchType=SIMPLE&language=sv&query=Development+of+a+Fall-Injury+Reducing+Flooring+System+in+Geriatric+Care+&af=[]&aq=[[]]&aq2=[[]]&aqe=[]&noOfRows=50&sortOrder=author_sort_asc&on.
60. Paka P, Karami G, Ziejewski M. Examination of brain injury under impact with the ground of various stiffness. In: Procedia Engineering. 2011. p. 409–14.
61. Redfern M, Moore P, Yarsky C. The influence of flooring on standing balance among older persons. Hum Factors J. 1997;39(3):445–55.
62. Rigby J, O’Connor M. Retaining older staff members in care homes and hospices in England and Australia: the impact of environment. Int J Palliat Nurs [Internet]. 2012;18(5):235–9. Available from: http://www.ncbi.nlm.nih.gov/pubmed/22885860.
63. Ryen L, Svensson M. Modelling the cost-effectiveness of impact-absorbing flooring in Swedish residential care facilities. Eur J Public Health [Internet]. 2015;26(3):407–11. Available from: http://eurpub.oxfordjournals.org/lookup/doi/10.1093/eurpub/ckv197.
64. Shimura Y, Okada H, Sakai T, Hayashi Y, Ohkawara K, Numao S, et al. Gait characteristics during walking on known tripping floor surfaces in older Japanese stroke patients. In: Journal of Aging and Physical Activity. Campaign, Illinois, USA: Human Kinetics Publ Inc; 2008. p. S46–7.
65. Simpson AHRW, Lamb S, Roberts PJ, Gardner TN, Grimley Evans J. Does the type of flooring affect the risk of hip fracture? Age Ageing. 2004;33(3):242–6.
66. Soangra R, Jones B, Lockhart TE. Effects of anti-fatigue flooring on gait parameters. In: Proceedings of the 54th Annual Meeting of the Human Factors and Ergonomics Society; 2010 Sep 27-Oct 1. San Francisco, California, USA. p. 2019-2022: SAGE Publications; p. 2019–22.
67. Soangra R, Lockhart TE. Determination of stabilogram diffusion analysis coefficients and invariant density analysis parameters to understand postural stability associated with standing on anti-fatigue mats. Biomed Sci Instrum. 2012;48.
68. Stephens JM, Goldie PA. Walking speed on parquetry and carpet after stroke: effect of surface and retest reliability. Clin Rehabil [Internet]. 1999;13(2):171–81. Available from: http://www.ncbi.nlm.nih.gov/pubmed/10348398.
69. Tibbitts GM. Patients who fall: How to predict and prevent injuries. Geriatrics [Internet]. 1996;51(9):24–8, 31. Available from: http://www.ncbi.nlm.nih.gov/pubmed/8814111.
70. Tideiksaar R. Mechanisms of falls in community residing older persons. Pride Inst J Long Term Home Health Care. 1993.
71. Tideiksaar R, Fletcher B. Keeping the elderly on their feet. Issues Sci Technol. 1989;5(3):78–81.
72. van der Woude LH, Geurts C, Winkelman H, Veeger HEJ. Measurement of wheelchair rolling resistance with a handle bar push technique. J Med Eng Technol. 2003;27(6):249–58.
73. Wahlström J, Östman C, Leijon O. The effect of flooring on musculoskeletal symptoms in the lower extremities and low back among female nursing assistants. Ergonomics. 2012;55(2):248–55.
74. Warren CJ, Hanger HC. Fall and fracture rates following a change from carpet to vinyl floor coverings in a geriatric rehabilitation hospital. A longitudinal, observational study. Clin Rehabil [Internet]. 2013;27(3):258–63. Available from: http://www.ncbi.nlm.nih.gov/pubmed/22843356.
75. Weaver TB, Laing AC. The influence of safety flooring on reactive stepping. National Falls Prevention Conference. 2016.
76. Willmott M. The effect of a vinyl floor surface and a carpeted floor surface upon walking in elderly hospital in-patients. Age Ageing [Internet]. 1986;15(2):119–20. Available from: http://www.ncbi.nlm.nih.gov/pubmed/3962760.
77. Wright AD, Heckman GA, McIlroy WE, Laing AC. Novel safety floors do not influence early compensatory balance reactions in older adults. Gait Posture. 2014;40(1):160–5.
78. Wright AD, Laing AC. The influence of novel compliant floors on balance control in elderly women - A biomechanical study. Accid Anal Prev. 2011;43(4):1480–7.
79. Wright AD, Laing AC. The influence of headform orientation and flooring systems on impact dynamics during simulated fall-related head impacts. Med Eng Phys. 2012;34(8):1071–8.
80. Wsynn, T., Riley, D., & Harris-Roberts, J. Ergonomic appraisal of the manual handling (push-pull) risk factors in areas using impact absorbing forces. Portsmouth (UK): Health and Safety Laboratory, University of Portsmouth.
81. Yarme J, Yarme H. Flooring and safety. Nurs Homes Long Term Care Manag. 2001;82.
82. Zacker C, Shea D. An economic evaluation of energy-absorbing flooring to prevent hip fractures. Int J Technol Assess Health Care [Internet]. 1998;14(3):446–57. Available from: http://www.ncbi.nlm.nih.gov/pubmed/9780531.
83. Center for Healthcare Environmental Management. Proper flooring: A critical measure for preventing slips and falls. Heal Hazard Manag Monit. 2003;16(10):1–5.
84. Rubberized flooring in long term care: Clinical effectiveness and cost-effectiveness. Rapid Response Report: Summary of Abstracts, Canadian Agency for Drugs and Technologies in Health. 2010.

**List of References of Records Included for Counts**

1. Bhan S, Levine I, Laing AC. Novel safety floors attenuate impact differentially across extreme BMI groups. In: Proceedings of the 17th Biennial Conference of the Canadian Society of Biomechanics; 2012 Jun 6-9 [Internet]. Burnaby, British Columbia, Canada; Available from: http://ocs.sfu.ca/csb-scb/index.php/csb-scb/2012/paper/viewFile/265/202.
2. Booth C, Gardner TN, Evans M, Simpson AHRW. The influence of floor covering on impact force during simulated hip fracture. In: Proceedings of the 1996 Annual Conference of the British Orthopaedic Research Society.
3. Bowers B, Lloyd J, Lee W, Powell-Cope G, Baptiste A. Biomechanical evaluation of injury severity associated with patient falls from bed. Rehabil Nurs [Internet]. 2005;33(6):253–9. Available from: http://www.ncbi.nlm.nih.gov/pubmed/19024240.
4. Callaghan JP, McGill SM. Impact forces from falling: Implications for low back injury. In: Proceedings of the 30th Annual Conference of the Human Factors Association of Canada. Mississauga, Ontario, Canada; 1998. p. 477–82.
5. Cameron ID, Murray GR, Gillespie LD, Robertson MC, Hill KD, Cumming RG, et al. Interventions for preventing falls in older people in nursing care facilities and hospitals. Cochrane Database Syst Rev. 2010;(1):CD005465.
6. Casalena JA, Badre-Alam A, Ovaert TC, Cavanagh PR, Streit DA. The Penn State Safety Floor: Part II--Reduction of fall-related peak impact forces on the femur. J Biomech Eng. 1998;120(4):527–32.
7. Cham R, Redfern MS. Influence of flooring on objective standing fatigue measures. In: Smith G, editor. Proceedings of the 23rd Annual Conference of the American Society of Biomechanics; 1999 [Internet]. Pittsburgh, Pennsylvania, USA; Available from: http://www.asbweb.org/conferences/1990s/1999/ACROBAT/025.PDF.
8. Abstract and commentary for: Choi Y. S., Lawler E., Boenecke C. A., Ponatoski E. R., Zimring C. M. Developing a multisystemic fall prevention model, incorporating the physical environment, the care process and technology: A systematic review. J Adv Nurs. 2011;67(12):2501–24.
9. Choi YS, Lawler E, Boenecke CA, Ponatoski ER, Zimring CM. Developing a multi-systemic fall prevention model, incorporating the physical environment, the care process and technology: A systematic review. Vol. 67, J Adv Nurs. 2011. p. 2501–24.
10. Abstract and Commentary for: Coussement J., De Paepe L., Schwendimann R., Denhaerynck K., Dejaeger E., & Milisen K. Interventions for preventing falls in acute and chronic care hospitals: A systematic review and meta-analysis. J Am Geriatr Soc. 2008;56(1):29–36.
11. Coussement J, De Paepe L, Schwendimann R, Denhaerynck K, Dejaeger E, Milisen K. Interventions for preventing falls in acute- and chronic-care hospitals: A systematic review and meta-analysis. Vol. 56, J Am Geriatr Soc. 2008. p. 29–36.
12. Cumming RG, Nevitt MC, Cummings SR. Epidemiology of hip fractures. Epidemiol Rev [Internet]. 1997;19(2):244–57. Available from: http://epirev.oxfordjournals.org/content/19/2/244.full.pdf+html?sid=1973fa77-e3c1-4ca8-9e79-751d755db4a7.
13. Currie LM. Fall and injury prevention. Annu Rev Nurs Res [Internet]. 2006;24:39–74. Available from: http://www.ncbi.nlm.nih.gov/entrez/query.fcgi?cmd=Retrieve&db=PubMed&dopt=Citation&list_uids=17078410.
14. Drahota A, Gal D, Windsor J. Flooring as an intervention to reduce injuries from falls in healthcare settings: An overview. Qual Ageing. 2007;8(1):3–9.
15. Drahota A, Gal D, Windsor J, Dixon S, Udell J, Ward D, et al. Pilot cluster randomised controlled trial of flooring to reduce injuries from falls in elderly care units: Study protocol. Inj Prev [Internet]. 2011;17(6):1–10. Available from: http://www.scopus.com/inward/record.url?eid=2-s2.0-81855164784&partnerID=40&md5=cde28a2c2b7bdf08f8c98a4c51dcd0b5.
16. Feldman, F., Korall, A., & Lachance, C. C. (2015). Physical and built environments care settings. In I. Pike, S. Richmond, L. Rothman, & A. Macpherson (Eds.), Canada injury prevention resource: An evidence-informed guide to injury prevention in Canada (pp. 167–177). Toronto, Ontario, Canada: Parachute.
17. Glinka MN, Karakolis T, Callaghan JP, Laing AC. Safety flooring to reduce the risk of fall-related injuries - Characterizing footfall deflections vs energy absorption during simulated impacts. In: Proceedings of the 17th Biennial Conference of the Canadian Society of Biomechanics; 2012 Jun 6-9. Burnaby, British Columbia, Canada; 2012.
18. Hanger HC, Hurley K, White A. Low impact flooring - does it reduce falls or injury. In: Proceedings of the 6th Biennial Australia and New Zealand Falls Prevention Conference. 2014. p. 65.
19. Hanger HC. Changing carpet to vinyl flooring: Effects on falls and fractures. In: Proceedings of the 4th Biennial Australia and New Zealand Falls Prevention Conference. 2010.
20. Harris DD, Detke LA. The role of flooring as a design element affecting patient and healthcare worker safety. Heal Environ Res Des J. 2013;6(3):95–119.
21. Juby AG. The challenges of interpreting efficacy of hip protector pads in fracture prevention in high-risk seniors. Clin Rheumatol. 2009;28(6):723–7.
22. Knoefel FD, Patrick L, Taylor J, Goubran R. Dual-Stiffness Flooring: Does it Reduce Fracture Rates Among Older Fallers? In: Journal of the American Geriatrics Society. Malden, Massachusetts, USA: Wiley-Blackwell; 2012. p. S129.
23. Korall AMB, Lachance CC, Russell CM, Johnson SI, Feldman F, Robinovitch SN, et al. Push Forces on Vinyl and Carpet for Conventional Wheeled and Motor-Driven Floor-Based Lifts Among Direct Care Staff in Long-Term Care. In: Proceedings of the 59th Annual Meeting of the Human Factors and Ergonomics Society. SAGE Publications; 2015. p. 1257.
24. Lachance, C. C., Korall, A. M. B., Russell, C. M., Johnson, S. I., Feldman, F., Robinovitch, S. N., & Mackey, D. C. (2015). Effects of Compliant Flooring Systems and Resident Weight on Hand Forces When Pushing Floor-Based Lifts and Wheelchairs Among Long-Term Care Staff. In Proceedings of the 59th Annual Meeting of the Human Factors and Ergonomics Society (Vol. 59, p. 1258).
25. Lachance, C. C., Korall, A. M.sss B., Russell, C. M., Johnson, S. I., Feldman, F., Robinovitch, S. N., & Mackey, D. C. (2015). Abstract for: Effects of compliant flooring systems and resident weight on hand forces when pushing floor-based lifts and wheelchairs among long-term care staff. In Proceedings of the 59th Annual Meeting of the Human Factors and Ergonomics Society.
26. Lachance CC, Laing AC, Robinovitch SN, Feldman F, Leung PM, Mackey DC. A randomized controlled trial to evaluate the effect of compliant flooring on fall-related injuries in long-term care: The flooring for injury prevention (FLIP) trial. In: Journal of Nutrition, Health and Aging. Seoul, South Korea; 2013. p. S498.
27. Lachance, C., Feldman, F., Robinovitch, S., & Mackey, D. C. (2012). Flooring for injury prevention. Infolink, 19(4), 12-13.
28. Lachance, C.C., Feldman, F., Robinovich, S., Mackey, D. C. (2013). Compliant flooring: A Potential Way of Reducing Injuries due to Falls. Osteoporosis Canada.
29. Lachance, C.C., Feldman, F., Robinovich, S., Mackey, D. C. (2015). Compliant flooring: A Potential Way of Reducing Injuries due to Falls. Osteoporosis Canada.
30. Lachance CC, Laing AC, Leung PM, Robinovitch SN, Feldman F, Mackey DC. Stakeholder investment and partnership pivotal for the success of a largescale randomized controlled trial in longterm care. In: Proceedings of the 14th Annual Conference of the Canadian Association on Gerontology; 2014 Oct 16-18. p. O17. Available: http://cag.conference-services.net/reports/template/onetextabstract.xml?xsl=template/onetextabstract.xsl&conferenceID=3732&abstractID=803829.
31. Laing ACT, Tootoonchi I, Robinovitch SN. Effect of floor stiffness on impact forces during falls on the hip. In: Proceedings of the 28th Annual Conference of the American Society of Biomechanics. Portland, Oregon, USA; 2004. Available: http://www.asbweb.org/conferences/2004/pdf/375.pdf.
32. Laing AC, Robinovitch SN. Design of low stiffness floors for preventing hip fractures in high risk environments: comparison of force attenuation and influence on balance. In: Proceedings of the 32nd Annual Conference of the American Society of Biomechanics. Ann Arbour, Michigan, USA; 2008. Available: <http://asbweb.org/conferences/2008/abstracts/555.pdf>
33. Loguidice C. Flooring affects risk of fall-related injuries in female nursing home residents. Am J Nurs. 2015;115(7):66.
34. Nanda U, Malone EB, Joseph A. Achieving EBD Goals through Flooring Selection & Design. 2012;83. Available from: http://www.healthdesign.org/sites/default/files/tandusflooringreport_final.pdf.
35. Nilson F. Fall-Related Injuries Amongst Elderly in Sweden Fall-Related Injuries Amongst Elderly in Sweden. Doctoral dissertation, Karlstad University Studies; 2014.
36. Oliver D, Connelly JB, Victor CR, Shaw FE, Whitehead A, Genc Y, et al. Strategies to prevent falls and fractures in hospitals and care homes and effect of cognitive impairment: systematic review and meta-analyses. BMJ. 2007;334(7584):82.
37. Oliver D, Healey F, Haines TP. Preventing falls and fall-related injuries in Hospitals. Vol. 26, Clinics in Geriatric Medicine. 2010. p. 645–92.
38. Quan X, Joseph A. Healthcare Environmental Terms and Outcome Measures: An Evidence-based Table of Contents. 2011;(November 2011):1–71.
39. Randall P, Burkhardt SSJ, Kutcher J. Exterior space for patients with Alzheimer’s disease and related disorders. Am J Alzheimers Dis Other Demen [Internet]. 1990;5(4):31–7. Available from: http://aja.sagepub.com/cgi/content/abstract/5/4/31\nhttp://aja.sagepub.com/cgi/reprint/5/4/31.pdf.
40. Robinovitch S. Technology for the prevention of fall-related injuries among older adults living in long-term care. Gerontechnology [Internet]. 2010;9(2):163–6. Available from: http://gerontechnology.info/index.php/journal/article/view/1187.
41. Robinovitch SN, Hsiao ET, Sandler R, Cortez J, Liu Q, Paiement GD. Prevention of falls and fall-related fractures through biomechanics. Exerc Sport Sci Rev. 2000;28(2):74–9.
42. Rowe J. Carpets can be used to reduce injury from falls. BMJ. 2002;324:1454.
43. SATECH. Highlights of Simon Fraser University study of SmartCells cushioned flooring. 2008.
44. Gulwadi GB, Calkins MP. The Impact of Healthcare Environmental Design on Patient Falls [Internet]. 2008. 33 p. Available from: https://www.healthdesign.org/sites/default/files/impact_of_healthcare_environment_design_on_patient_falls.pdf.
45. Shojania KG, Duncan BW, McDonald KM, Wachter RM, Markowitz a J. Making health care safer: A critical analysis of patient safety practices. Evid Rep Technol Assess (Summ) [Internet]. 2001;2001(43):i – x, 1–668. Available from: http://www.ncbi.nlm.nih.gov/pubmed/11510252.ss
46. Tideiksaar R. Fall prevention in the home. Top Geriatr Rehabil. 1987;3(1):57–64.
47. Tideiksaar R. How flooring can reduce fall risk and injury. Ext Care Prof News. 2007;24–7.
48. Abstract and Commentary for: Tse T. The environment and falls prevention: Do environmental modifications make a difference? Aust Occup Ther J. 2005;52(4):271–81.
49. Tse T. The environment and falls prevention: Do environmental modifications make a difference? Aust Occup Ther J. 2005;52(4):271–81.
50. Udell JE. Fall and injury prevention interventions: An exploration using three complementary methodologies. Doctoral dissertation, University of Portsmouth; 2013.
51. Vieira ER, Freund-Heritage R, da Costa BR. Risk factors for geriatric patient falls in rehabilitation hospital settings: a systematic review. Clin Rehabil [Internet]. 2011;25(9):788–99. Available from: http://www.ncbi.nlm.nih.gov/pubmed/21504956.
52. Wallis SJ, Campbell GA. Preventing falls and fractures in long-term care. Rev Clin Gerontol [Internet]. 2011;21(4):346–60 15p. Available from: https://acces.bibl.ulaval.ca/login?url=https://search.ebscohost.com/login.aspx?direct=true&db=rzh&AN=104587623&lang=fr&site=ehost-live.
53. Wright A. Novel Compliant Flooring Systems from Head to Toes: Influences on Early Compensatory Balance Reactions in Retirement-Home Dwelling Adults and on Impact Dynamics during Simulated Head Impacts. Master’s Thesis, University of Waterloo; 2011.
54. Wright AD, Laing AC. The influence of novel compliant flooring systems on impact dynamics during simulated impacts to the back of the head. In: Proceedings of the 23rd Biennial Congress of the International Society of Biomechanics; 2011 Jul 3-7. Brussels, Belgium; p. 271–5.
55. Weaver, T. B., & Laing, A. C. (2016). The influence of safety flooring on reactive stepping. Watch Your Step National Fall Prevention Conference. Calgary, Alberta, Canada.
56. Wright AD, Laing AC. Towards the reduction of fall-related injury risk: Novel compliant floors do not influence rate of balance control responses in older women. In: Proceedings of the 16th Biennial Conference of the Canadian Society of Biomechanics; 2010 Jun 9-12 [Internet]. Kingston, Ontario, Canada; Available from: http://health.uottawa.ca/biomech/csb/Archives/CSB 2010 proceedings_all papers.pdf.
